# Report of Foreign Relations Committee

**Published:** 1900-10

**Authors:** 


### REPORT OF FOREIGN RELATIONS COMMITTEE.

CONSIDERABLE space is given in this number to the report of this committee. It is interesting and, while open to criticism in some points, exhibits an earnest effort in the right direction.

There is a mistake in the statement that it "was adopted" at the National Association of Dental Faculties. If that were the motion, as originally made, it was a parliamentary error. The foundation rule of the Association is that all motions, resolutions, or reports affecting colleges must lie over for one year before final action. There are a number of suggestions in this report that affect the interests of a number of the colleges, members of the Association, hence the report could not be and was not adopted. It can be taken up next year at the meeting in Milwaukee, and considered in detail, but until this is done and the official report received, members of the Association and the foreign advisory committees cannot be required to consider it in their rulings.

